# *CHRNA5* and *CHRNA3* polymorphism and lung cancer susceptibility in Palestinian population

**DOI:** 10.1186/s13104-018-3310-0

**Published:** 2018-04-02

**Authors:** Basim Mohammad Ayesh, Rami Al-Masri, Abdalla Assaf Abed

**Affiliations:** 1 Department of Laboratory Medical Sciences, Alaqsa University, Gaza, Palestine P.O. Box 4051,; 2 Central Laboratory, Ministry of Health, Gaza, Palestine; 3 Biology Department, Islamic University of Gaza, Gaza, Palestine

**Keywords:** s16969968, rs1051730, Lung cancer, Gaza strip, ASP-PCR, *CHRNA5*, *CHRNA3*, nAChR

## Abstract

**Objective:**

The genetic polymorphism (rs16969968 in *CHRNA5*, and rs1051730 in *CHRNA3* genes) were recently shown to be associated with risk of LC. The aim of this study is to elucidate whether they predispose Palestinian individuals to lung cancer, and how is this related to smoking.

**Results:**

Frequency of the rs16969968-A allele was significantly higher in the case group (36.7%) than in normal controls (17.5%; *P* = 0.022; OR = 6.83 for AA and 2.81 for AG genotypes). The frequency of rs1051730-T allele was also significantly higher in the case group (46.7%) than in the control group (22.5%; *P* = 0.001; OR = 2.20 for TC and 13.22 for TT genotypes). Frequency of rs16969968-A allele was higher in smokers (29.1%) than nonsmokers (15.7%) regardless of lung cancer; similarly, frequency of rs1051730-T allele was also higher in smokers than in smokers (46.7% vs 22.5%, respectively). The higher the proportion of the risk allele (rs16969968-A and rs1051730-T), the higher the mean number of daily consumed cigarettes (*P* = 0.006). Carrying rs16969968-A and/or rs1051730-T alleles results in an increased risk to lung cancer probably by increasing the individual’s tendency for heavy smoking. The allelic frequency of the rs16969968-A and rs1051730-T alleles among normal Palestinian controls is similar to different populations worldwide.

**Electronic supplementary material:**

The online version of this article (10.1186/s13104-018-3310-0) contains supplementary material, which is available to authorized users.

## Introduction

Lung cancer (LC) has been, for decades, the leading cause of male cancer related death both in developing and developed countries [1]. Recently, it has surpassed breast cancer as the leading cause of cancer death in females in developed countries, probably because of the lately spread of tobacco epidemic in females [2]. Both active and passive smoking are well known risk factors for lung cancer [3–9]. Between 80 and 90% of lung cancer are attributed to smoking [10, 11]. Nevertheless, the majority of smokers don’t develop LC during their life, most likely as a result of genetic polymorphism. Research has pointed the role of the person’s genetic makeup in growing his/her likelihood of becoming attached to cigarettes as well as in increasing susceptibility to lung cancer [12–15]. Independent genome-wide association studies (GWAS) identified two single nucleotide polymorphisms (SNPs) in the genes for nicotinic acetylcholine receptor (rs16969968 in *CHRNA5*, and rs1051730 in *CHRNA3* genes) to be consistently associated with risk of LC [16, 17]. Those polymorphisms are most likely increasing the risk of LC via increasing the individual’s dependence to nicotine [18–22].

About 22.5% of Palestinian individuals (≥ 18 years) are smokers [23]. LC is overall the third leading cause of death (9.8% of reported cases), and is one of the major causes of morbidity among Palestinian population [24]. Therefore, in this study we aimed at determining the frequency of the SNPs (rs16969968 in *CHRNA5*, and rs1051730 in *CHRNA3*) and elucidating their possible role in risk for nicotine dependence and lung cancer among the Palestinian population.

## Main text

### Methods

#### Study design and population

The present study is a case control study with convenience sample. Cases were recruited from all LC patients managed in the oncology departments of Al-Shifa Hospital (n = 17) and European Gaza Hospital (n = 13) during the period from February 2014 to June 2014. Patients were excluded if their LC was a metastasis originating from other organs. The diagnosis of lung cancer was previously confirmed by the diagnostic tools available in the managing hospitals. The majority of cases were males (93.3% males and 6.7% females). The mean age of lung cancer cases was 61.4 ± 9.1 years (range 45–80 years).

The control group comprised 60 participants (30 smoker and 30 nonsmokers) matched to the cases in gender (71.7% males and 8.3% females) and age (mean = 60.4 ± 7.9 years; range 43–80 years).

#### Sample collection and genomic DNA extraction

About 5 ml peripheral blood were collected from each case and control in EDTA-anti-coagulated vacutainers. Genomic DNA was extracted from peripheral lymphocytes using the QIAamp DNA Blood Mini kit (Qiagen, Germany) according to the manufacturer instructions.

#### Genotype determination with allele-specific primer (ASP) PCR

Allele-specific (AS) primers (Additional file 1) were designed using the Web-based Allele-Specific PCR assay (WASP) online software [25] and purchased from (Hylabs, Israel). Additional mismatches were intentionally introduced at the penultimate base of the AS primers to increase specificity as described earlier [26].

Two separate 20 μl-reactions were performed for each SNP, one for the wild-type allele and the second for the variant allele. All amplifications were carried out in the presence of 1× GoTaq Green Master Mix (Promega, USA), 50 ng genomic DNA, 0.5 µM common sense primer and 0.5 µM wild-type specific antisense or mutant-specific primers. The temperature profile for the SNP: rs16969968 included an initial denaturation at 95 °C for 5 min, followed with 38 cycles of 95 °C for 30 s, 58 °C for 30 s and 72 °C for 45 s. A final 10 min extension step was performed at 72 °C. The temperature profile for the SNP: rs1051730 was the same except for annealing temperature which was performed at 59 °C for 30 s. To validate the results, 10% randomly selected samples were re-genotyped and the results were concordant.

The amplification products were resolved by 2% agarose gel at 100 V for 50 min in 1× TBE buffer. The gels were stained with 0.5 μg/µl ethidium bromide and UV-visualized in a gel documentation system.

#### Data analysis

Data were collected from the patients including their age, gender, smoking status, number of cigarettes per day, type of cancer, years of smoking, period of quit smoking and others. The data was analyzed by the Statistical Package for the Social Sciences (SPSS; version 19). The student t test was applied to detect significant mean differences and the Pearson’s χ^*2*^ test to detect distribution differences of categorical variables. Statistical significance was set at a threshold of P = 0.05 or less. The Hardy–Weinberg equilibrium was examined for the distribution of genotypes in each group using a goodness of fit χ^*2*^ test.

### Results

#### ASP-PCR genotyping

**Fig. 1:**
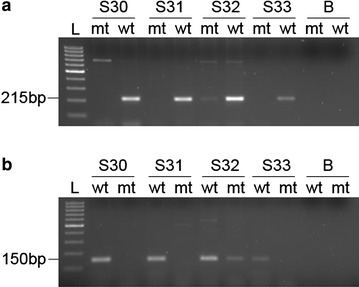
A representative agarose gel electrophoresis of ASP-PCR. **a** The SNP rs16969968 and **b** the SNP rs1051730 were genotyped with two separate reactions for each sample: one using wildtype-specific primer (**wt**) and the other using mutant-specific primers (**mt**). *L* 100 bp DNA ladder and *B* blank water sample. Samples (**S30**, **S31**, and **S33**) are wildtype homozygous and sample **S32** is heterozygous for both SNPs

The ninety subjects enrolled in the study were successfully genotyped for the rs16969968 and rs1051730 by allele-specific primers PCR (Fig. 1). Repeated genotyping of a number of samples consistently gave concordant results.

#### Distribution of CHRNA5 (c.1192G > A)/rs16969968

The distribution of cases and controls by genotype of rs16969968 is shown in (Additional file 2). The alleles are in Hardy–Weinberg equilibrium (*P* = 0.6165), and the overall frequency of rs16969968-A is calculated to be 23.9% (Additional file 2). It is higher in the case group (36.7%) than in the control group (17.5%). Distribution of the genotypes among cases and controls is statistically significant (*P* = 0.022). Frequency of rs16969968-AA homozygotes and rs16969968-GA heterozygotes is higher in cases than in controls. Homozygosity and heterozygosity for the minor allele increases susceptibility to lung cancer when compared to the wild type genotype (OR = 6.83 and 2.81, respectively; Additional file 3).

#### Distribution of CHRNA3 (c.65C > T)/rs1051730

Alleles of rs1051730 are also in Hardy–Weinberg equilibrium (*P* = 0.887). The overall frequency of rs16969968-T allele is 31.1% (Additional file 2). The allelic frequency of the susceptibility allele is higher in the case group (46.7%) than in the control group (22.5%). Frequency of the rs1051730-TT and rs1051730-CT genotypes is significantly higher in cases than in controls (*P* = 0.001; Additional file 2). Carriage of the rs1051730-T allele in a homozygous or heterozygous genotype increases the risk for lung cancer when compared to the wild type genotype (OR = 3.05; Additional file 3).

#### Smoking status

Only four lung cancer cases were nonsmoker cases, while the rest 26 were smokers. Few patients have quitted smoking after being diagnosed and thus were considered smokers. The amount of cigarettes smoked per day by lung cancer patients and smoker controls was not significantly different (*P* = 0.823; Additional file 4). On the other hand, the average period of smoking was found to be significantly higher in lung cancer cases (39.04 ± 12.7 years) compared to smoker controls (28.0 ± 10.5 years) (*P* = 0.001; Additional file 4).

#### Relationship between smoking and genotype in lung cancer

Differences in distribution of the rs16969968 genotypes between the smoker cases and smoker controls was not statistically significant (*P* = 0.272; Additional file 5). Analysis of statistical significance could not be applied to compare non-smoker case (n = 4) and non-smoker control (n = 30) because of the small sample size. The allelic frequency of the rs16969968-A allele is higher in smokers (29.1%) than nonsmokers (15.7%), regardless of lung cancer.

On the other hand, differences in distribution of the rs1051730 genotypes between smoker cases and smoker controls was statistically significant (*P* = 0.015; Additional file 5). Frequency of rs1051730-T allele is higher in smokers (26.6%) than nonsmokers (18.3%). regardless of lung cancer.

Table 1 shows that the higher the proportion of rs16969968-A allele or rs1051730-T allele, the higher the mean number of daily consumed cigarettes (*P* = 0.006 for both alleles).

**Table 1:** Effect of minor alleles on smoking dependence

Genotype	No. cigarettes/day (mean ± SD)	*P* value
*CHRNA5* (c.1192G > A) rs16969968		
GG	25.54 ± 11.6	0.006
GA	36.04 ± 12.2	
AA	40.0 ± 15.81	
*CHRNA3* (c.65C > T) rs1051730		
CC	24.86 ± 12.97	0.006
CT	33.00 ± 10.36	
TT	39.17 ± 15.62	

The average duration of smoking was not significantly different between the different genotypes of rs16969968 and rs1051730 genotypes (*P* = 0.402 and 0.320, respectively; Table 2).

**Table 2:** Effect of genotype on mean smoking duration

	Duration (years) mean ± SD	*P* value
*CHRNA5* (c.1192G > A) rs16969968		
GG	35.4 ± 12.4	0.402
GA	30.6 ± 13.6	
AA	31.8 ± 10.7	
*CHRNA3* (c.65C > T) rs1051730		
CC	35.8 ± 13.1	0.320
CT	30.6 ± 12.9	
TT	35.8 ± 10.3	

### Discussion

In this study, we have developed and applied an ASP-PCR technique for genotyping the rs16969968 and rs1051730 SNPs at the *CHRNA5/CHRNA3* locus. The worldwide frequency of rs16969968-A allele ranges from 1% in certain populations such as African and Japanese to more than 47% in others such as European populations [27]. The frequency in our normal control individuals falls in the middle 17.5%. The rs16969968-A allele frequency is higher in the case group (36.7%). The distribution of rs16969968 genotypes among cases and controls is statistically significant (*P* = 0.022), supporting the potential role of rs16969968-A in risk of LC (OR = 6.83 for rs16969968-AA and OR = 2.81 for rs16969968-AG). Like previously reported data, this allele may be predisposing to LC independently from smoking [28–30].

The allelic frequency for the T-allele of the SNP rs1051730 in the control group is 22.5%. The overall worldwide frequency of rs1051730-T allele is 16.8%, ranging from 2.7% in east Asia to 47.7% in Iberian population of Spain [27]. Similar to rs16969968, frequency of rs1051730-T allele is higher in the case group, and the distribution of rs1051730 genotypes between cases and controls is statistically significant (*P* = 0.001). The rs1051730-TC and -TT genotypes seem to confer the individual susceptible to LC (OR = 2.20 and 13.22, respectively). These findings are parallel to previously published data which demonstrated that rs1051730-T allele is risk-conferring for the development of lung cancer in different ethnic groups [31–34]. These data also suggest an independent role for rs1051730-T allele in predisposition to lung cancer apart from smoking. The rs1051730 polymorphism was suggested to modify susceptibility to lung cancer among Chinese Han population by a smoking-independent manner [35, 36]. It was also suggested to function as a genetic modifier for the risk of developing lung adenocarcinoma in nonsmoking Chinese females [37].

The postulation that both SNPs increase the risk of LC independently from smoking comes from Additional file 4, in which the mean number of daily consumed cigarettes was not significantly different between the smoker cases and smoker controls (*P* = 0.823). Rather, the duration of smoking was far more important in predicting lung cancer risk (*P* = 0.001; Additional file 4) [38–40]. In this regard, Table 2 shows that the genotype of both SNPs does not significantly influence the duration of smoking.

The allelic frequency of rs16969968-A and rs1051730-T susceptibility alleles was calculated in smokers and nonsmokers regardless of LC. The frequency of rs16969968-A allele was higher in smokers (29.1%) than nonsmokers (15.7%). The frequency of rs1051730-T allele was also higher in smokers than in smokers (46.7% vs. 22.5%, respectively). Furthermore, Table 1 shows that the higher the proportion of the risk allele (rs16969968-A and rs1051730-T), the higher the mean number of daily consumed cigarettes (*P* = 0.006). Accordingly, we may consider homo- or heterozygosity for the rs16969968-A and rs1051730-T allele predisposing for smoking dependence. In support of this assumption, carriers of the rs16969968-A allele were reported to have significantly higher serum nicotine levels, and to correlate with Fagerström test results for Nicotine Dependence [41]. Furthermore, higher smoking intensity was reported in rs1051730-CT heterozygote and rs1051730-TT homozygote smokers compared with wild-type smokers [42, 43].

Tobacco smoking increases the risk of at least 17 classes of human cancer probably by increasing the somatic mutation load [44]. Nicotine and other tobacco components may contribute to lung cancer by modifying the gene expression to induce epithelial to mesenchymal transition (EMT) [45–47]. Nicotine can also contribute to proliferation in LC through the activation of nicotinic acetylcholine receptors (nAChRs) [48]. Therefore, we may conclude that each of the rs16969968-A and rs1051730-T alleles increases the individual’s dependence on tobacco smoking, which in turn confers him/her susceptible to LC. This conclusion supports previously reported association of rs16969968-A with LC risk largely via tobacco exposure [22, 49–51]. Lips, and colleagues demonstrated that association between rs16969968-A and LC risk was unchanged after adjusting for smoking (OR = 1.30 VS smoking adjusted OR = 1.27) [52]. Similar to our results, an increased nicotine level indicates an increased risk of LC with each additional copy of the rs1051730 and rs16969968 risk allele [51, 53, 54]. A recent meta-analysis concluded that the rs16969968-A predicts delayed smoking cessation and an earlier age of lung cancer diagnosis [55].

In conclusion, carrying one or the other of the risk alleles rs16969968-A and rs1051730-T results in an increased risk to lung cancer probably by increasing the individual’s tendency to smoke more and at an earlier age. The allelic frequency of the rs16969968-A and rs1051730-T alleles among normal Palestinian controls falls within the worldwide range in different populations.

## Limitations

The main limitation of the present study is the small sample size, which limited our ability to detect moderate interactions especially in the stratified analyses. Larger studies are needed in the future to confirm our findings in the Palestinian population. In addition, lack of information and absence of properly organized patient record at the managing hospital limited access to the patients.

## Additional files

**Additional file 1.** List of AS-primers for each SNP.

**Additional file 2.** Genotype distribution of the SNP rs16969968 and the SNP rs1051730 among cases and controls.

**Additional file 3.** rs16969968 and rs1051730 genotypes and risk of lung cancer.

**Additional file 4.** Cigarettes consumption in smoker cases VS smoker controls.

**Additional file 5.** Genotype and minor allele distribution among smoker cases and controls.

## Abbreviations

nAChR: nicotinic acetylcholine receptors
LC: lung cancer
GWAS: genome-wide association studies
SNP: single nucleotide polymorphism
ASP-PCR: allele-specific primer PCR
OR: odds ratio
