# Supplementary material for: CHRNA5 and CHRNA3 polymorphism and lung cancer susceptibility in Palestinian population
Source: BMC Res Notes. 2018 Apr 2;11:218. doi: 10.1186/s13104-018-3310-0 (PMC5879790; doi:10.1186/s13104-018-3310-0)
Supplement: Supplementary file 1 — Additional file 1. List of AS-primers for each SNP. [file 13104_2018_3310_MOESM1_ESM.pdf]

Additional file 1: List of AS-primers for each SNP

Gene<br>SNP	Sequance (5'→3')‡	Description	Amplicon Size (bp)
CHRNA5<br>(c.1192G>A)<br>rs16969968	CTTGTAATGTAGCGAATAGAAg**<u>C</u>**	Wild-type antisense	215
	CTTGTAATGTAGCGAATAGAAg**<u>T</u>**	Mutant antisense	
	CGCTATCAACATTCATCATC	Common sense	
CHRNA3<br>(c.65C>T)<br>rs1051730	TTGTACTTGATGTCGTGTTg**<u>G</u>**	Wild-type antisense	150
	TTGTACTTGATGTCGTGTTg**<u>A</u>**	Mutant antisense	
	ACTGTACCATGAAGTTCGGT	Common sense	

‡ the underlined bold letter indicates the wild-type and variant specific nucleotide. The lowercase letter indicats intentionally introduced mismatches at the penultimate base.
