# Supplementary material for: CHRNA5 and CHRNA3 polymorphism and lung cancer susceptibility in Palestinian population
Source: BMC Res Notes. 2018 Apr 2;11:218. doi: 10.1186/s13104-018-3310-0 (PMC5879790; doi:10.1186/s13104-018-3310-0)
Supplement: Supplementary file 2 — Additional file 2. Genotype distribution of the SNP rs16969968 and the SNP rs1051730 among cases and controls. [file 13104_2018_3310_MOESM2_ESM.pdf]

Additional file 2: Genotype distribution of the SNP rs16969968 and the SNP rs1051730 among cases and controls.

***CHRNA5* (c.1192G>A) rs16969968**				
**Genotype**	**Case (n=30)**	**Control (n=60)**	**Total (n=90)**	***P*-value**
**GG**	12 (40%)	41(68.3%)	53 (58.9%)	0.022
**GA**	14 (46.7%)	17 (28.3%)	31 (34.4%)	
**AA**	4 (13.3%)	2 (3.4%)	6 (6.7)	
**Frequency of the minor allele (A)**	36.7%	17.5%	23.9%	
***CHRNA3* (c.65C>T) rs1051730**				
**Genotype**	**Case (n=30)**	**Control (n=60)**	**Total (n=90)**	***P*-value**
**CC**	9 (30.0%)	34 (56.7%)	43 (47.8%)	0.001
**CT**	14 (46.7%)	25 (41.7%)	39 (43.3%)	
**TT**	7 (23.3%)	1 (1.6%)	8 (8.9%)	
**Frequency of the minor allele (T)**	46.7%	22.5%	30.5%	
