# Supplementary material for: CHRNA5 and CHRNA3 polymorphism and lung cancer susceptibility in Palestinian population
Source: BMC Res Notes. 2018 Apr 2;11:218. doi: 10.1186/s13104-018-3310-0 (PMC5879790; doi:10.1186/s13104-018-3310-0)
Supplement: Supplementary file 3 — Additional file 3. rs16969968 and rs1051730 genotypes and risk of lung cancer. [file 13104_2018_3310_MOESM3_ESM.pdf]

Additional file 3: rs16969968 and rs1051730 genotypes and risk of lung cancer.

Genotype		OR	95%CI	*P*-value
***CHRNA5* (c.1192G>A) rs16969968**	GA/AA VS GG	3.23	1.30-8.05	0.010
	GA VS GG	2.81	1.08-7.32	0.029
	AA VS GG	6.83	1.11-41.97	0.041
***CHRNA3* (c.65C>T) rs1051730**	CT/TT VS CC	3.05	1.20–7.75	0.015
	CT VS CC	2.20	0.82–5.91	0.113
	TT VS CC	13.22	2.33–74.9	0.001
