# Supplementary material for: CHRNA5 and CHRNA3 polymorphism and lung cancer susceptibility in Palestinian population
Source: BMC Res Notes. 2018 Apr 2;11:218. doi: 10.1186/s13104-018-3310-0 (PMC5879790; doi:10.1186/s13104-018-3310-0)
Supplement: Supplementary file 4 — Additional file 4. Cigarettes consumption in smoker cases VS smoker controls. [file 13104_2018_3310_MOESM4_ESM.pdf]

Additional file 4: Cigarettes consumption in smoker cases VS smoker controls.

		**Smoker Case**	**Smoker Control**	***P*-value**
**Number of Cigarettes/day**	Mean ±SD	30.2±13.2	31.0±12.5	0.823
**Duration (Years)**	Mean ±SD	39.04 ±12.7	28.0 ±10.5	0.001
