# Supplementary material for: CHRNA5 and CHRNA3 polymorphism and lung cancer susceptibility in Palestinian population
Source: BMC Res Notes. 2018 Apr 2;11:218. doi: 10.1186/s13104-018-3310-0 (PMC5879790; doi:10.1186/s13104-018-3310-0)
Supplement: Supplementary file 5 — Additional file 5. Genotype and minor allele distribution among smoker cases and controls. [file 13104_2018_3310_MOESM5_ESM.pdf]

Additional file 5: Genotype and minor allele distribution among smoker cases and controls.

**Genotype**		**Smoker Case**	**Smoker Control**	***P*-value**
CHRNA5 (c.1192G>A) rs16969968	GG	10 (38.5%)	18 (60%)	0.272
	GA	13 (50%)	10 (33.3%)	
	AA	3 (11.5%)	2 (6.7%)	
Frequency of the minor allele (A)		36.7%	23.3%	
CHRNA3 (c.65C>T) rs1051730	CC	7 (26.9%)	14 (46.7%)	0.015
	CT	13 (50%)	16 (53.3%)	
	TT	6 (23.1%)	0	
Frequency of the minor allele (T)		41.7%	26.7%	
